# Photoaging Mobile Apps: A Novel Opportunity for Smoking Cessation?

**DOI:** 10.2196/jmir.4792

**Published:** 2015-07-27

**Authors:** Titus J Brinker, Werner Seeger

**Affiliations:** ^1^Universities of Giessen and Marburg Lung Center (UGMLC)Member of the German Center for Lung Research (DZL)Justus-Liebig-University GiessenGiessenGermany

**Keywords:** tobacco prevention, tobacco cessation, photoaging, mobile phone apps, apps, app, smartphones

Most smokers start smoking during their early adolescence with the idea that smoking is glamorous; the problems related to lung cancer, vascular disease, and chronic pulmonary disease are too far in the future to fathom. In contrast, most adolescents view their image in a mirror as an important component of their personal life. A recent randomized controlled trial by Burford et al published in the *Journal of Medical Internet Research* demonstrated an increased quit rate of 21% in 18-30-year-old young adults by the help of photoaging desktop programs, in which an image is altered to predict future appearance [1]. Furthermore, the photoaging software has been shown to increase the motivation of 14-18-year-old females to quit [2]. However, the investigated programs only reach a small audience and are not freely available.

We took advantage of the widespread availability of mobile phones and adolescents´ interest in appearance to develop a free mobile phone app which requires the user to take a self portrait (ie, a selfie), which is then displayed by the photoaging software as four images: consequences of (non-)smoking one pack a day for a year (Figure 1) or 15 years (Figure 2). Afterwards, the app explains the visual results and offers many sharing options with family and friends. By this means, the social network of the user may also be informed about the various beauty reducing effects of smoking, potential health consequences, and learn about the app.

The underlying aging algorithms take into account the user’s current age and are based on publications showing an increased risk for acne and pale skin due to declined capillary perfusion (after one pack-year), as well as connective tissue changes and wrinkles in the longer term (after 15 pack-years) [3,4].

The app has been installed on over 50,000 Android and 27,000 iOS mobile phones within seven months after its release in Germany (10/27/2014 to 4/26/2015). As mobile phone use in Germany declines with age, the largest fraction of the app’s users are assumed to be 30 years or younger.

Based on the publication from Burford et al, it is reasonable to speculate that the app could motivate smokers to quit. Taking into account that the smoking prevalence in the general German population is approximately 25% (approximately 19,250 of the 77,000 app users were smokers), about 4000 users (21%) would have quit after using the app. Further research is needed to investigate the effectiveness of app-based photoaging interventions to increase quit rates and to prevent smoking initiation.

**Figure 1:**
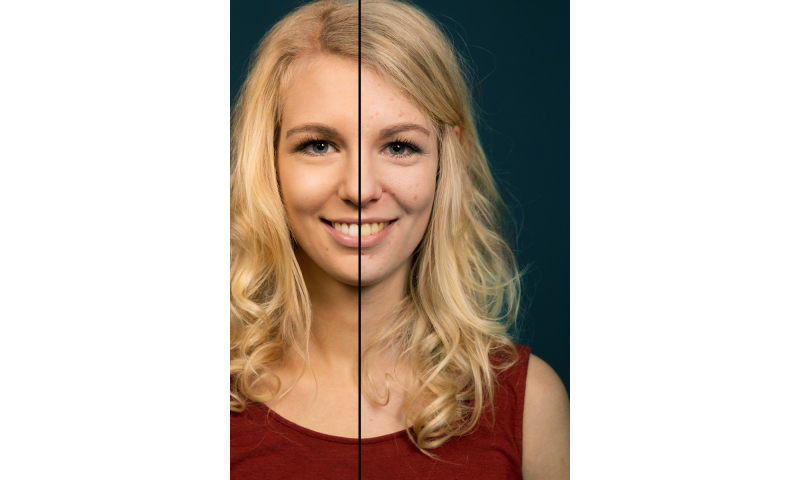
Photoaged image of a 17 year old woman showing the consequences of smoking one pack a day for one year (vs. non-smoking).

**Figure 2:**
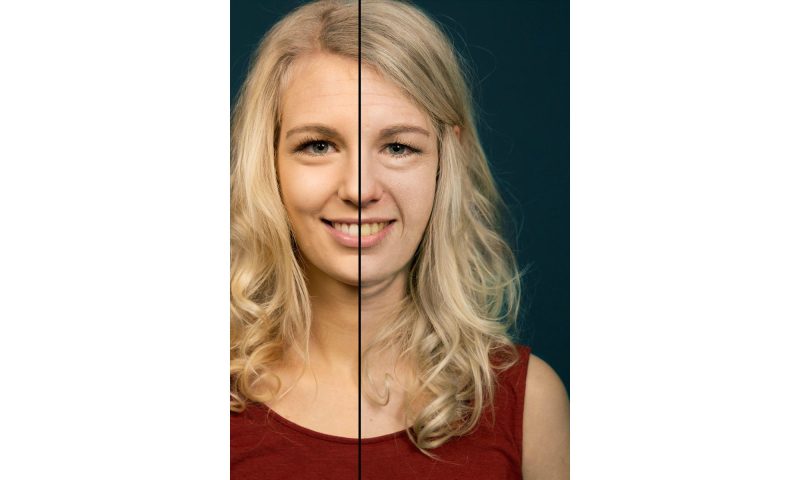
Photoaged image of a 17 year old woman showing the consequences of smoking one pack a day for 15 years (vs non-smoking).

## References

[1] Burford Oksana, Jiwa Moyez, Carter Owen, Parsons Richard, Hendrie Delia (2013). Internet-based photoaging within Australian pharmacies to promote smoking cessation: randomized controlled trial. J Med Internet Res.

[3] Weiss Carine, Hanebuth Dirk, Coda Paola, Dratva Julia, Heintz Margit, Stutz Elisabeth Zemp (2010). Aging images as a motivational trigger for smoking cessation in young women. Int J Environ Res Public Health.

[3] Grady D, Ernster V (1992). Does cigarette smoking make you ugly and old?. Am J Epidemiol.

[4] Schäfer T, Nienhaus A, Vieluf D, Berger J, Ring J (2001). Epidemiology of acne in the general population: the risk of smoking. Br J Dermatol.

